# High energy density storage, antifungal activity and enhanced bioimaging by green self-doped heteroatom carbon dots

**DOI:** 10.1186/s11671-023-03910-9

**Published:** 2023-10-23

**Authors:** Mohd Abdullah Sheikh, R. S. Chandok, Khan Abida

**Affiliations:** 1 Bhagwant University, Ajmer, Rajasthan 305004 India; 2 Sri Guru Tegh Bahadur Khalsa College, Jabalpur, India; 3 Government Degree College for Women Anantnag, Srinagar, India

**Keywords:** Heteroatom-doped N-CDs, Microwave irradiation, Ni/N-CDs, Supercapacitance, Cladosporium cladosporioides

## Abstract

Self-heteroatom-doped N-carbon dots (N-CDs) with a 2.35 eV energy gap and a 65.5% fluorescence quantum yield were created using a one-step, efficient, inexpensive, and environmentally friendly microwave irradiation method. FE-SEM, EDX, FT-IR, XRD, UV–VIS spectroscopy, FL spectroscopy, and CV electrochemical analysis were used to characterise the produced heteroatom-doped N-CDs. The graphitic carbon dot surface is doped with heteroatom functional groups such (S, P, K, Mg, Zn) = 1%, in addition to the additional passivating agent (N), according to the EDX surface morphology and the spontaneous heteroatom doping was caused by the heterogeneous chemical composition of pumpkin seeds. These spontaneous heteroatom-doped N-CDs possess quasispherical amorphous graphitic structure with an average size of less than 10 nm and the interplaner distance of 0.334 nm. Calculations utilising cyclic voltammetry showed that the heteroatom-doped N-CDs placed on nickel electrodes had a high specific capacitance value of 1044 F/g at a scan rate of 10 mV/s in 3 M of KOH electrolyte solution. Furthermore, it demonstrated a high energy and power density of 28.50 Wh/kg and 3350 W/kg, respectively. The higher value of specific capacitance and energy density were attributed to the fact that the Ni/CDs electrode material possesses both EDLC and PC properties due to the sufficient surface area and the multiple active sites of the prepared N-CDs. Furthermore, the heteroatom N-CDs revealed the antifungal action and bioimaging of the "Cladosporium cladosporioides" mould, which is mostly accountable for economic losses in agricultural products. The functional groups of nitrogen, sulphur, phosphorus, and zinc on the surface of the CDs have strong antibacterial and antifungal properties as well as fluorescence enhanced bioimaging.

## Introduction

Photoluminescence, super capacitive, anti-bacterial, bioimaging, photocatalytic and much more capabilities may be seen in carbon dots which are created via pyrolysis, that incorporates the breakdown, dehydrolysis, and carbonisation of carbonaceous materials. Xu and colleagues unintentionally discovered carbon NT'S in mid-2004 [1] while examining it, which further got their name as "Carbon Dots" in 2006 [2], when Sun and his colleagues verified their synthesis process and associated structure. Carbon dots (C-dots), a luminous carbon nanomaterial with a size smaller than 10 nm, constitutes a member of the family of carbon nanomaterials. The high quantum yield (HQY) and controlled emission wavelength of these quasi-spherical particles are just a few of their numerous benefits. C-dots' superior biocompatibility, ease of surface modification, high photostability, low cytotoxicity, and chemical inertness creates an opportunity to try out novel planning strategies as well as discover novel application domains. It is now utilised for a variety of purposes, including the creation of multicoloured light-emitting diodes (LEDs), energy generation and storage, live cell imaging, in vivo research, photocatalytic degradation, drug administration, fluorescence sensing, and many more [3–21].

The top-down perspective approach uses laser ablation, arc discharge, and electrochemical release and constrains larger carbon composites—such as graphite, massive graphene, synthetic carbon nanofibers, and activated industrial charcoal—to minute nanometric C-dots are less beneficial than bottom-up strategies like pyrolysis, hydrothermal technique, convection method, solvothermal method, and ultrasonic method as the former often employs challenging operational procedures like acid solutions, electrical discharges, expensive equipment, and tedious procedures [22–39]. However, every CD featured a unique distribution of emission trap sites, regardless of the synthesis technique. Due to the ease in attachment of various functional groups, their surfaces can be modified, to achieve a wider range of potential applications than semiconductor quantum dots and fluorescent biological groups, including biological imaging, energy storage, chemo-sensing, environmental control, diagnostic testing, and photocatalytic degradation. The exploration of living and artificial carbon sources for C-Dots is centered on two different types of carbonaceous raw materials. In terms of sustainability, biomass precursors have the potential to be used in the production of carbon dots due to their affordability, accessibility, environmental friendliness, and capacity to arrange biological waste [40–44]. De facto, they supply a lot of spontaneous ingredients, such as lignin, cellulose, lignocellulose, carbohydrates, proteins, triglycerides, and naturally occurring elements like iron, phosphorus, zinc, magnesium, and manganese, which serve as strong self-dopants and increase the quantum yield efficiency. A lot of biomass resources, namely soybean milk (2.6%) [45], vegetation (6.2%) [46], strawberry extract juice (6.3%) [47], ground coffee (3.8%) [48], spice ginger (13.4%) [49], Lychee seed (10.3%) [50], Peanut shell (9.91%) [51] and dairy milk (12%) [52] (Quantum yield in parenthesis), have been utilised as raw precursors in the hydrothermal preparation of N-CDs.

Doping heteroatoms such as N, B, P, S, and other atoms into the carbon nano-structure can enhance the electro-catalytic performances of these nano-carbonaceous materials. For example nitrogen-doping into carbon nanomaterials including CNTs, graphene, and GCDS have found many advantages over non-doped carbon nanomaterials like creating a "healing" effect. In addition, the conjugation system acquires an extra pair of electrons from N, increasing the number of redox process active sites and also incorporating heteroatoms in the carbon network, allowing the insertion of electro-catalytic active sites with little alteration to the conjugation length [53]. These positive traits have attracted a lot of interest in the fields of super capacitor energy storage, bioimaging, and antibacterial compounds. In contrast to batteries or fuel cells, supercapacitors (SCs) are crucial for efficient electrochemical energy storage because of their high power density, rapid charge–discharge rate, prolonged life cycle, and simple working mechanism [53, 54]. The two widely used techniques of electric double-layer capacitance (EDLC) and pseudo-capacitance (PC) are subsequently utilised to attain these traits. A fast oxidation–reduction process is used by the pseudo-capacitance mechanism [55–58]. PC electrodes are frequently made from activated carbon-based materials like carbon nanotubes (CNT), carbon dots (CDs), and graphene because of their high porosity, low cost, chemical and physical stability, and ease of production from sustainable resources [59–62]. Incorporating pseudo-capacitive features and putting together carbon-based materials with large surface areas and suitable pore sizes, such as metal oxides, metal hydroxides, and polymers, or substances with surface functionalised with oxygen or nitrogen have been successful in achieving substantially greater capacitance values [63–65] and these energy storage devices often referred to as hybrid SCs.

In an effort to achieve immediate, prolonged, and severe examination of bacteria and fungi, green C-dots have been looked into it. For instance, Carica papaya Juice-derived C-dots were examined for imaging of bacterial (Bacillus subtilis) and fungal cells (Aspergillus aculeatus) as well as demonstrated the intracellular wavelength optimised emission. Furthermore, aforementioned team investigated pomegranate-derived C-dots for scanning Fusarium avenaceum and Pseudomonas aeruginosa cells [66] and found similar coloured cytoplasmic fluorescence. For the imaging of E. coli, Das et al. [67] used luminous c-dots generated from grammes.

The surface functional groups on carbon dots also exhibit antibacterial capabilities, such as using quaternised ammonia CDs have ability to kill gram-positive bacteria with specificity [68, 69]. Moreover, CDs have a tremendous amount of antiviral potential. In 2020, Kotta et al. examined the possible usage of CDs against several corona virus types and talked about the potential for employing CDs to fight COVID19 [70]. Au@CDs was used in a different investigation to show the ability to fight against the fungus Candida albicans. [71] If contrasted with conventional and innovative antimicrobial agents, CDs had greater biocompatibility and reduced biotoxicity. Synthetic parameters like the type of solvent used during the reaction process, reaction time, PH, reaction temperature, carbon source, level of nitrogen content, and/or particle size, as well as heteroatom doping, all appear to have a greater impact on tuning the emission of CDs.

 In this study, green synthesised blue emitting primarily nitrogen, spontaneous trace elements like phosphorus, sulphur, zinc, potassium, and magnesium-doped CDs (G-CDs) were made from pumpkin seeds and urea as nitrogen precursor in hydrogen peroxide solvent system using a microwave carbonisation method and without adding any toxic material. These CDs exhibit a broad absorption band between 250 and 550 nm, with minor peaks at 254 and 278 nm and a significant peak at 327 nm that are associated to the π—π* transition of the C=C bond and the n-π* transition of the C=O and C=N, C=S bonds, respectively. Substantial fluorescence appeared as a result of excited multi-state energy trapping, which gave rise to this peak at 327 nm. Under natural light, the aqueous solution of synthesised heteroatom carbon dots' was clear and yellow, but under UV light (400 nm), it shined a vivid blue color. The highest fluorescence intensity was seen at 462 nm when excited at 380 nm. The carbon dots' fluorescence emission spectra displayed a typical excitation-dependent behaviour with a quantum yield of (65.50%).

In order to boost the performance of the supercapacitor, a complex of the produced heteroatom-doped N-CDS and carbon black electrode materials was loaded onto a nickel foam (NF) substrate. Superior specific capacitance values of 1044 F g^−1^ at a scan rate of 10 mV s^−1^ were confirmed by the experimental results. A higher wattage power density of 4500 (W/kg) and an energy density of 29.36 (Wh/kg) are also shown by the statistics. As a result, heteroatom-doped N-CDs and carbon black composites become suitable options for utilisation as blended supercapacitor electrode materials.

Prior research has demonstrated that carbon dots doped with sulphur, phosphorus, zinc, and nitrogen have potent antimicrobial effects against gram-positive, gram-negative, and a variety of fungal species. Therefore, our study (heteroatom-doped N-CDs) exhibits a super-resolution imaging and extraordinary antifungal activity of cladosporium cladosporioides mould, which is responsible for economic losses in various farming industries.

## Experimental section

### Materials, synthesis and characterisation

Pumpkin seeds, urea (99.99%) pure, H_2_O_2_, NaOH, de-ionised water throughout the whole process, pH papers, syringe filer 210 nm, dialysis tube (10,000 mwco), quinine sulphate, H_2_SO_4_,, HCl, Nickel foam, carbon black, PVDF binder, ethanol, cladosporium spp fungus sample.

### Methodology and synthesis

Spontaneous heteroatom-doped N-carbon dots were prepared from pumpkin seed kernel and seed shell in a ratio of 2:1 by adding 3 g of kernel and 2 g of shell along with 3 g of urea as the nitrogen source. First pumpkin seeds were purchased from the local market, washed with acetone to remove organic waste or dust and dried in hot oven air at 60 ^°^C for 1 h. Following the drying process the pumpkin kernel and shells were separated from the whole seeds and then ground separately to fine powder. The material was taken in above proportion in 50 ml H_2_O_2_ solvent along with 3 gms of urea and mixed well with magnetic stirrer for 30 min at room temperature. After this, the slurry mixture was kept in a 200 ml conical funnel and treated with microwave irradiation at 900 W for 8 min until a black solid residue was left behind then 10 ml of de- ionised water was mixed to dissolve the solid and finally filtered using filter paper pore size 11 µm. The sample was then centrifuged at 4000 rpm for 10 min to remove bigger molecules and un-reacted precursor material. Separate all the hereto-atom N-doped carbon dots from the centrifuged solution by employing a 0.21 µm Teflon syringe filter and finally remove the ions and small molecules by dialysis employing a 10,000 MWCO dialysis membrane for 6 h in 1 L de- ionised water to obtain a pure solution of heteroatom N-doped carbon dots, the pH was to kept 5 by adding NaOH and finally dried at 60 degree to obtain a final self-heteroatom N-CDs powder.

### Preparation of working electrode for CV measurements

The synthesised heteroatom-doped N-CDs were combined at Weight % values of 90, 5, and 5 with carbon black and a binder (PVDF). The combination was then dispersed in 5 ml of an ethanol and clean water solution. After a brief period of stirring, the mixture was sonicated at 40 kHz for 30 min to obtain a well-dispersed precipitate. In order to get rid of contaminants and stains, the nickel foam was submerged in 1 M HCl before being extensively rinsed with DI water. The slurry was smeared carefully on this Nickel Foam, letting it dry for 15 min, subsequently sintered for 30 min at 150 ^0^C. The final working electrodes were created with a loading of 1 mg/cm^−2^ of working material to conserve it for further processing.

Preparation of cladosporium cladosporioides fungus culture.

By using the transparent polymer tap method, Cladosporium cladosporioides samples were extracted from dried bean pods. The pure culture was then cultivated on PDA (Synthetic Potato-Dextrose-Agar) and cultured for seven days at 25 °C in the dark.

### Characterisation

Field emission scanning electron microscopy (FE-SEM) and energy dispersive X-ray spectroscopy (EDX) were performed on a SUPRA 55 VP- 4132 CARL ZEIS with an accelerating voltage of 10.0 kV to study the morphology and elemental composition of the heteroatom-doped N-CQDs nanocomposite. Fourier transform infrared spectrometry (FTIR) was recorded on a spectrum Two™ FT-IR spectrometer to investigate the chemical bonding in the heteroatom-doped N-CQDs sample. The crystalline phase of the sample was identified by X-ray diffraction (XRD) using Rigaku’s new D/teX Ultra-250 instrument under Cu-kβ radiation in the 2ϴ range of 10°–80°. The UV–Vis absorption spectra of the heteroatom-doped N-CQD solution were obtained using a double beam UV–Vis spectrometer; Cat no BE/CI/SP/DB-S-04. The Photoluminescence (PL) spectra’s of the heteroatom-doped N-CQDs are obtained by a fluorescence spectrophotometer F-7000. The electro chemical measurements were performed using a potentiostat in an electrochemical cell with three electrodes immersed in an electrolyte solution.

## Results and discussion

### Characterisation of self-heteroatom-doped N-carbon dots

**Fig. 1:**
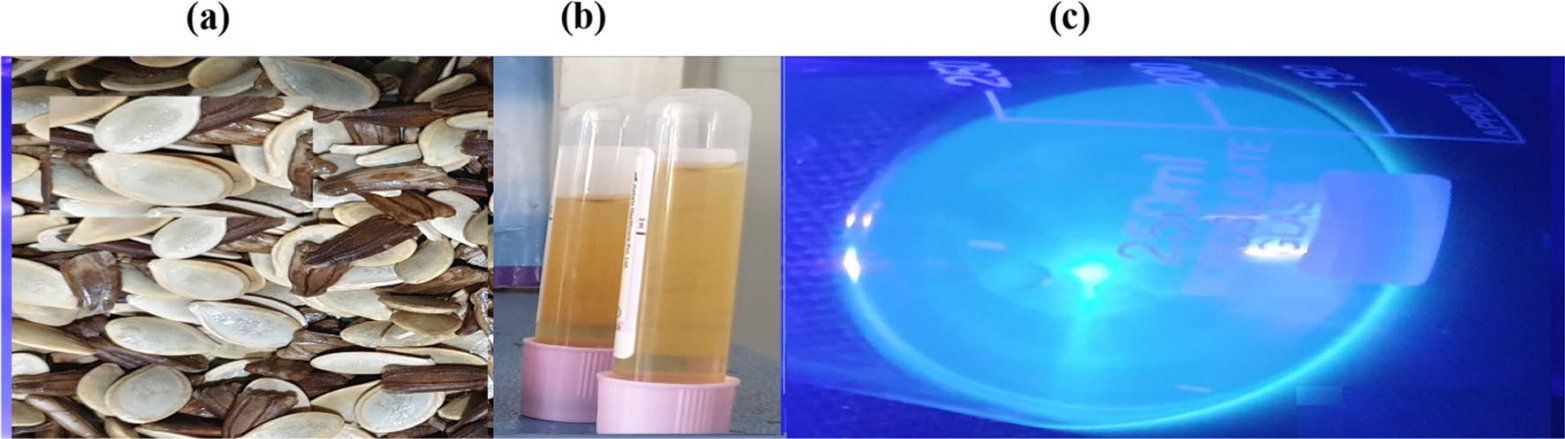
**a** Pumpkin seed precursor **b** Carbon dots under visible light and **c** Carbon dots under UV light

In this work, pumpkin seeds are used as a carbon source to create self-heteroatom-doped N-carbon dots, as shown in Fig. 1a. Furthermore, the "urea" is used as a nitrogen source for surface passivation or changes their surface states. After the microwave irradiation reaction, a light brown solution was produced that shined brightly blue when exposed to UV light (390 nm), as illustrated in Fig. 1b and c, respectively.

The sum of pumpkin seeds, microwave power, and reaction time were all gradually optimised with the amount of urea fixing set at 3 g in order to get a high fluorescence quantum yield. The QY of these CDs first rose and then dropped as the pumpkin seed content increased until it reached its peak at 3 gm. The number of pumpkin seeds at 3 gm with a quantum yield of 65.5% was then optimised with a microwave irradiation power of 900 W and reaction duration of 8 min. Only a sufficiently enough temperature and long enough reaction time can ensure that the precursor is adequately carbonised to produce CDs and a high QY. In contrast, a considerably higher reaction temperature and longer reaction time may destroy the produced emissive sites and caused a reduction in QY.

### Morphology and size distribution

**Fig. 2:**
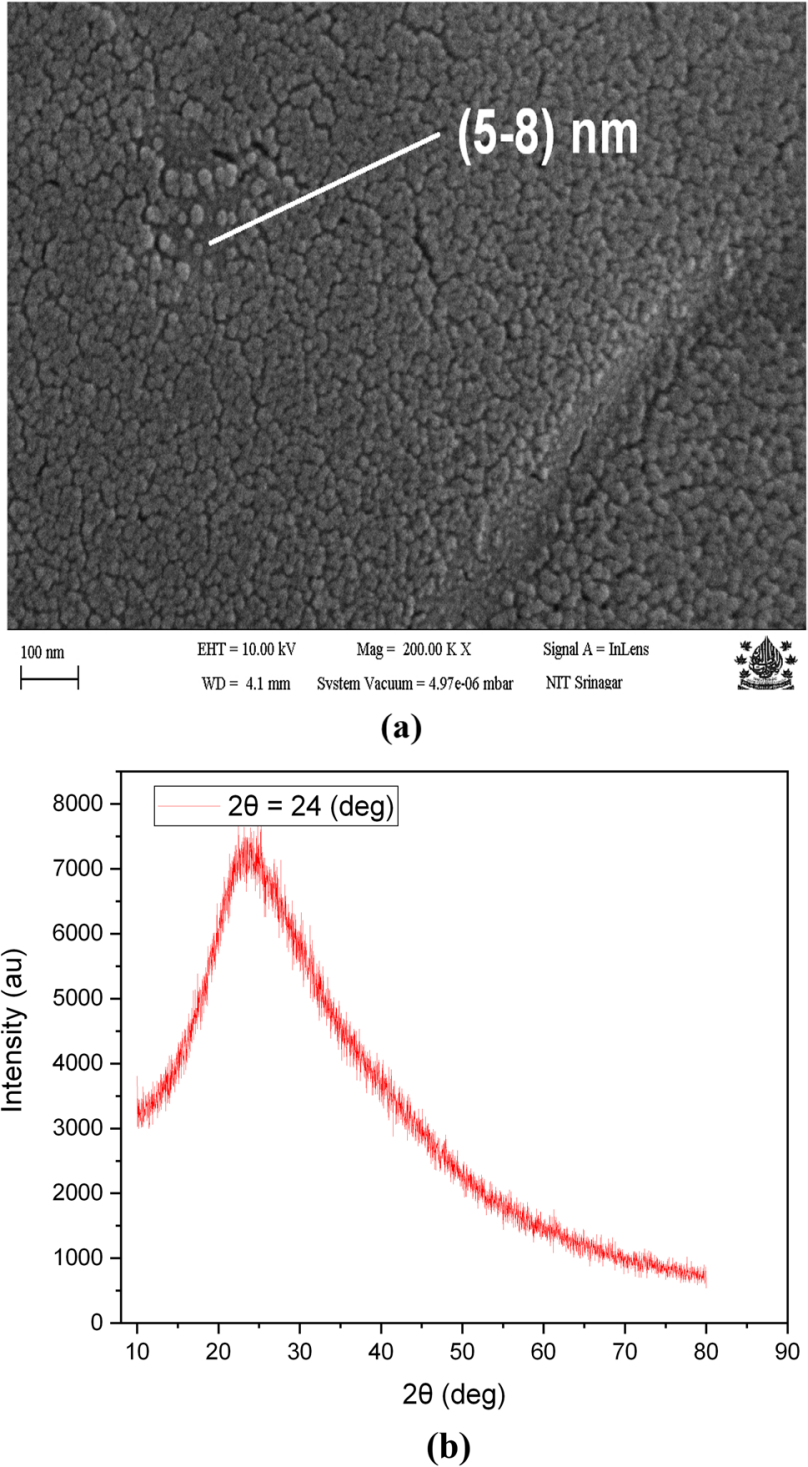
**a** SEM mage showing carbon dots with nanometer size distribution on left side **b** XRD graph of heteroatom N-CDs showing amorphous morphology on right side

The morphology and microstructure of the self-heteroatom-doped N-CDs, were explored using SEM as shown in Fig. 2a. These heteroatom-doped N-CDs have a relatively uniform size distribution with a size distribution of 4 nm–8 nm, with slight agglomeration. As seen in Fig. 2b, the X-ray diffraction pattern of N-CDs has a clearly defined wide peak at 20°–40°, which denotes the production of the very tiny nanoparticles. The produced N-CDs had a large peak around 24° when exposed to Cu_K-beta (1.39 nm) radiation, revealing disordered carbon atoms with a (002) plane and a 0.334 nm interlayer spacing of hexagonal (hcp) lattice structure in the core structure.

### Structural and elemental analysis

**Fig. 3:**
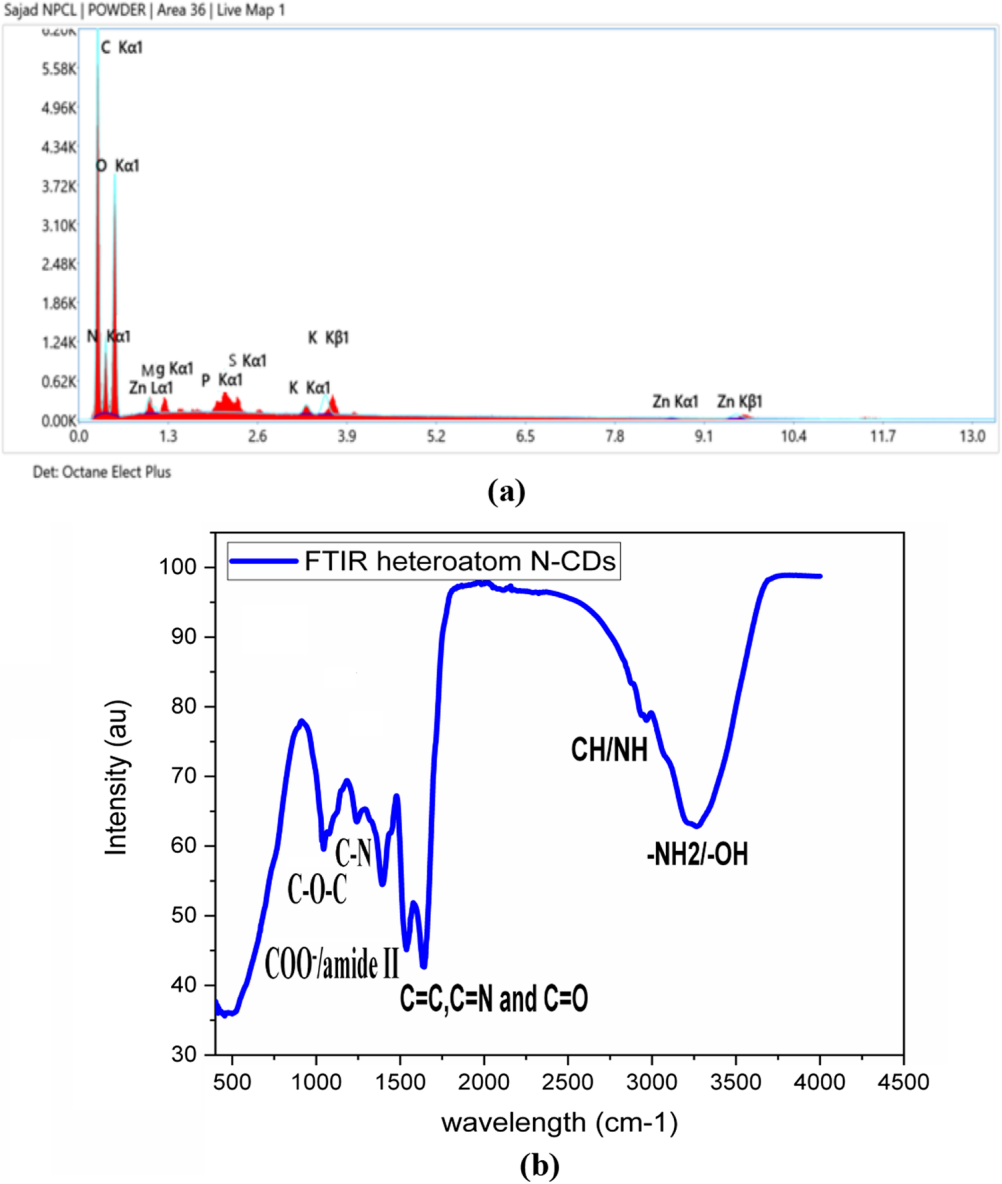
**a** FTIR spectra of synthesised self-heteroatom-doped N-carbon dots on left side **b** EDX spectra of self-heteroatom-doped N-carbon dots for elemental mapping on right side

The as-prepared N-CDs have a variety of surface functional groups with C and O, as shown by the FTIR spectrum in Fig. 3a. The stretching vibrations of O–H and N–H are represented by the absorption bands between 3100 and 3500 cm^−1^, respectively. The stability and hydrophilicity of the N-CDs in various solvent regimes were mostly attributed to these groups. The C-H vibrational stretching is what causes the tiny absorption peak at 2950 cm^−1^. These findings show that the self-heteroatom-doped N-CDs have amine, hydroxyl, and carboxylic functional groups on their surface. Furthermore, the two peaks at (1537 and 1393 cm^−1^) belongs to asymmetric and symmetric stretching of carboxylate groups COO-/amide II that are likely continuing to exist on the CD surface as the derivatives of the precursor. The absorption band at (1642 cm^−1^) corresponds to C=C/C=N/C=O stretching vibration. C-N and starching of C-O bonds are represented by 1240 and 1043 cm-1, respectively. In addition to that, the small bands around 1050 and 500 cm^−1^ show a fever self-heteroatom-doping elemental composition which is verified by EDX, as shown below.

Furthermore, the elemental analysis of the synthesised CDs was conducted using Energy-dispersive X-ray spectroscopy (EDX). The study's results (Fig. 3b) suggested that the following weight percentages were present: 48.3% C, 32.6% O, 18% N, 0.2% Mg, 0.2% P, 0.2% S, 0.3% K, and 0.2% Zn.

### Optical properties

**Fig. 4:**
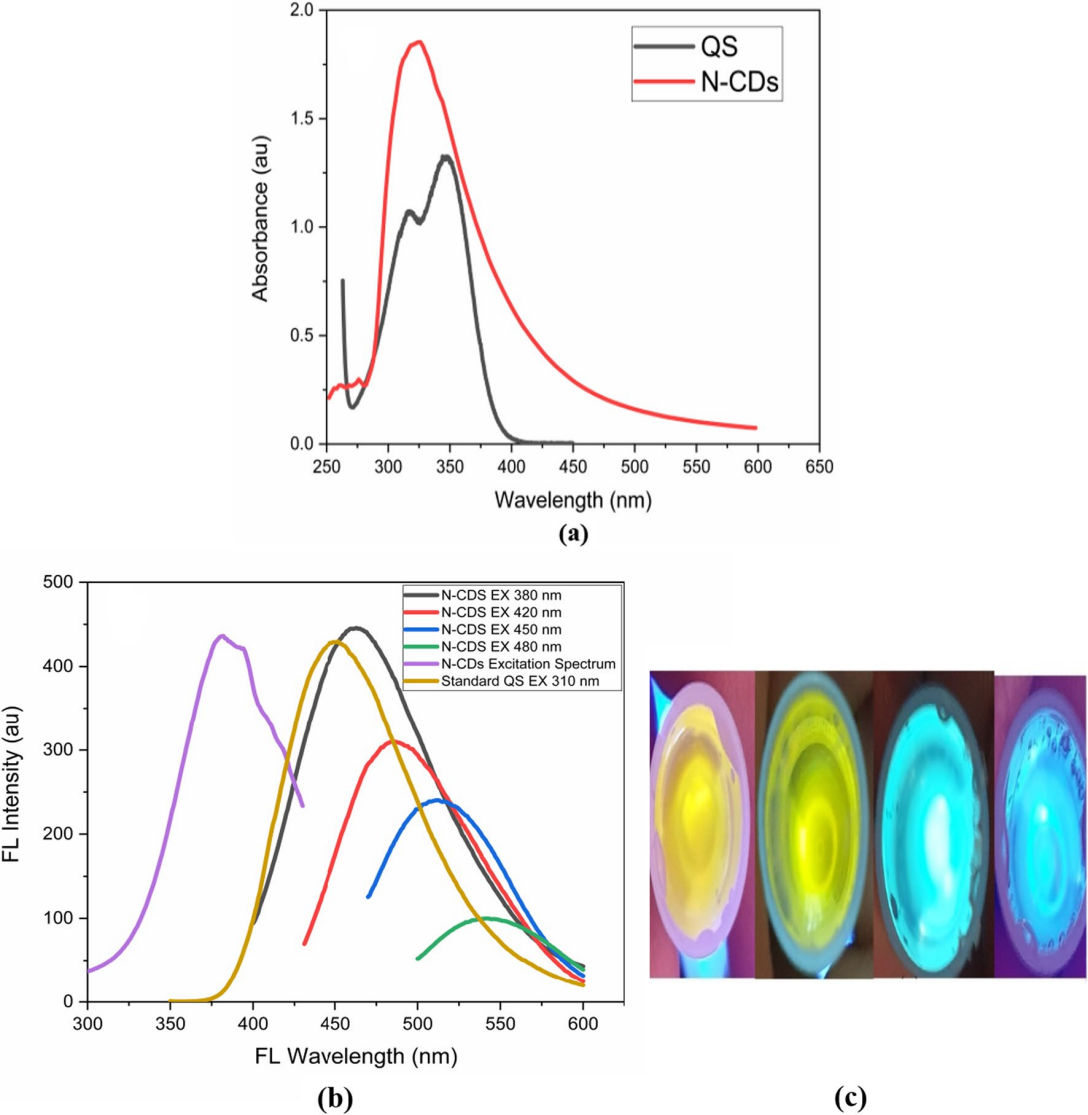
**a** Absorption Spectra of Heteroatom-doped N- Carbon dots and reference standard Quinine sulphate **b** Excitation and Emission spectra of synthesised N-doped carbon dots along with emission spectra of reference standard (QS) (**c**) N-CDs absorbed various wavelengths and emitted different colors

Absorption and PL spectroscopy are used to show the synthesised heteroatom-doped N-CDs' good optical characteristics. The prepared N-doped CDs showed a broad absorption region with a wavelength range of 254–550 nm. The small absorption peaks at 254 and 278 nm are caused by the π-π* transaction of the C=C interaction, while the other major peak at 327 nm is attributed to the n-π* transition of C=N, C=O in the N-doped CDs, as seen in Fig. 4a.The N-doped C-dots' emission wavelength was red-shifted from 462 to 542 nm while their excitation wavelengths was ranged from 380 to 480 nm. Furthermore, as demonstrated in (Fig. 4b), the N-doped CDs solution's ideal excitation and emission wavelengths were at 380 and 462 nm, respectively. Under various absorbed wavelengths, the N-doped CD aqueous solution fluoresced blue, cyan, green and yellow colors and can be seen clearly in Fig. 4c.

The PL excitation spectrum of the synthesised N-doped CDs was examined in order to learn more about their optical characteristics. The spectrum exhibited normal excitation wavelength dependency, as shown in Fig. 4b, and was redshifted when stimulated at longer wavelengths. It has been hypothesised that the N-doped CDs' behaviour is a result of their various sizes or the presence of various emissive sites on their surfaces. As a result, it has been recommended that these N-doped CDs possess super-capacitive as well as antifungal and bioimaging property. This excitation-dependent emission feature of carbon dots has also been discovered in earlier papers. The fluorescence intensity remained unaltered without photobleaching under continuous illumination at 380 nm for five hours, supporting their moderately high photostability.

**Fig. 5:**
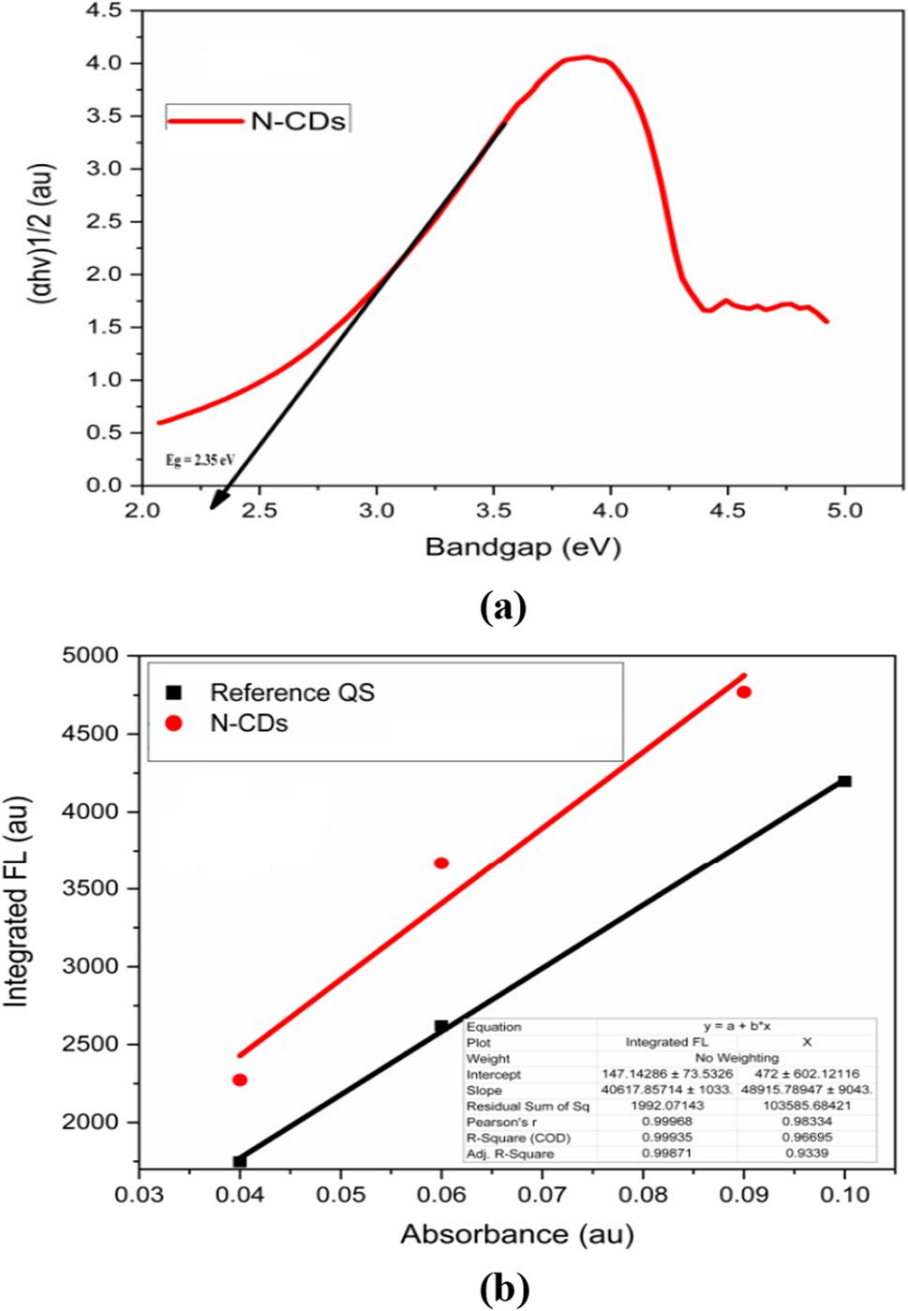
**a** Tauc plot of N-CD’s for energy gap determination **b** Integrated fluorescence intensity of Reference standard Quinine sulphate and N-CD’s vs absorbance along with their linear fits

The direct bandgap energy of these synthesised N-CDs is low approximately, 2.35 eV (or 527 nm), and was estimated via the Tauc plot technique from the absorption data as shown in Fig. 5a. The overall quantum efficiency of the produced N-CD’s was approximately 65.50% in accordance with the reference standard quinine sulphate dispersed in 0.1 mol of H_2_SO_4_ (absorption and emission spectra’s as already shown in Fig. 4a, b, respectively. At absorbance values of less than 0.1 of both sample and reference, excited at 380 and 310 nm, respectively, and the acquired data was plotted and linear fitted to get the gradients of each sample as shown in Fig. 5b. The Grad_N-CDs_ = 48,955 and Grad_Ref_ = 40,617.

Equation (1) was used to determine the QY of N-CDs,

1 $${\mathbf{QY}} \, = \, {\mathbf{QY}}_{{{\mathbf{Ref}} \, *}} \left( {{\mathbf{k}}/{\mathbf{k}}_{{{\mathbf{Ref}}}} } \right)_{*} \left( {{{\varvec{\upeta}}}/{{\varvec{\upeta}}}_{{{\mathbf{Ref}}}} } \right)^{{\mathbf{2}}}$$

In this equation, QY stands for the quantum yield, k stands for the gradient of the plot with integrated fluorescence intensity as a function of absorbance, η is the solvent's refractive index (1.33), and (Ref) for the reference (quinine sulphate) (1.33) approximately.

### Electrochemical measurements

**Fig. 6:**
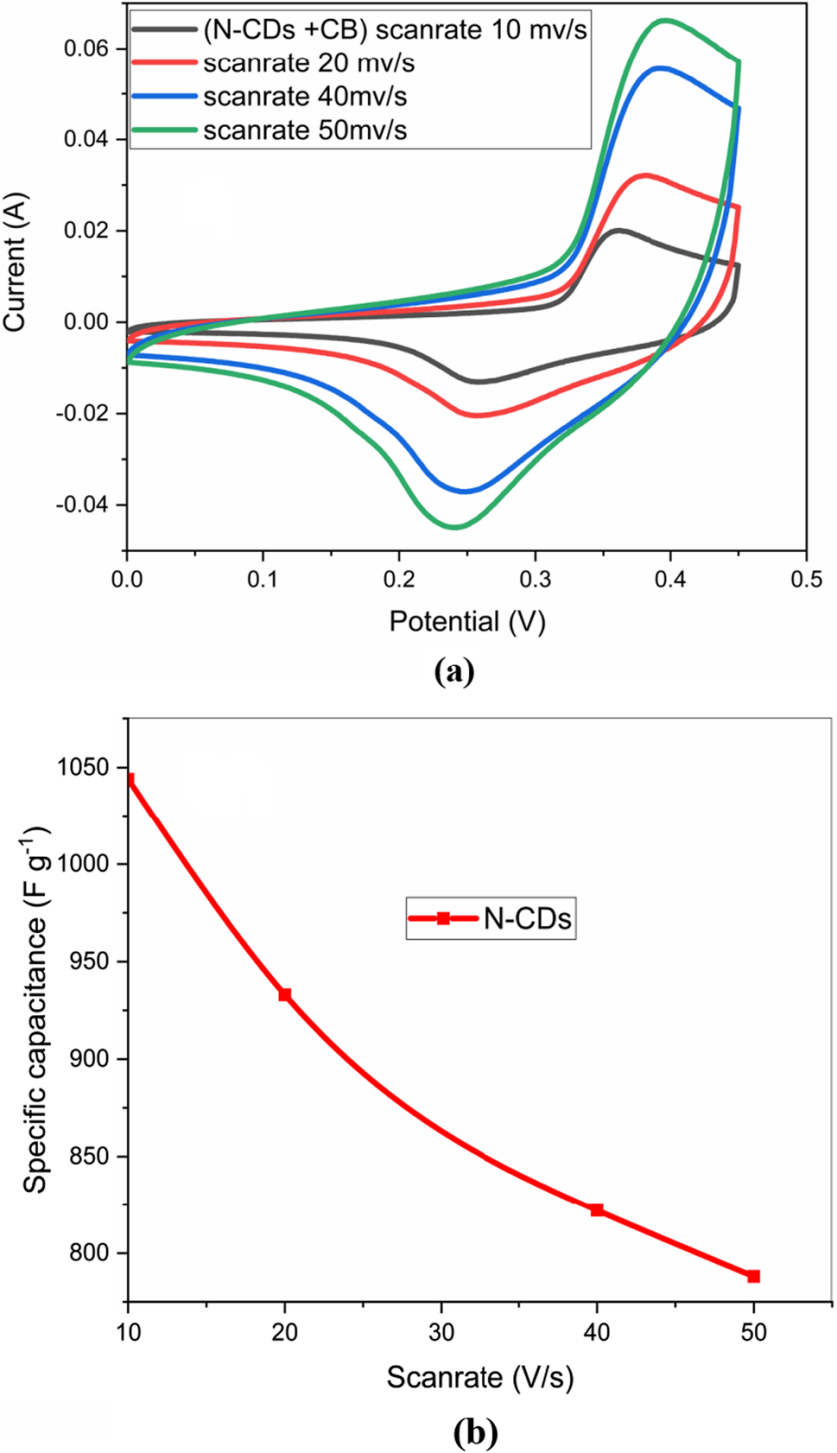
**a** CV curves of (Ni/N-CDs) Hybrid SC material under different scan speeds **b** Achieved specific capacitances at various scan speeds

In a three electrode cell with platinum as the counter electrode, Ag/AgCl as the reference electrode, and the manufactured Ni/N-CDs as the working electrode in 3 M of KOH, Cyclic voltammetry measurements were used to analyse the created working electrode. As can be seen in Fig. 6a, the CV measurements were carried out in the potential range of (0- 4.5)V at scan speeds of 10, 20, 40, and 50 (mV/s).

The results showed good oxidation and reduction peaks because of the faradic processes and also somewhat resembled a rectangular shape that demonstrated the EDLC behaviour of this NF/N-CDs working electrode material. This confirms the hybrid capacitive property of the heteroatom-doped N-CDs deposited on the nickel foam. The area of the CV curve is directly proportional to the specific capacitance value. As the scan rate increased, the oxidation peak of the heteroatom-doped N-CDs shifted to a higher potential, and the reduction peak shifted to a lower potential.

This can be explained by the relatively slow pace of the electrochemical reactions at higher scan speeds, which suggests the electron transfer mechanism may be reversible or nearly reversible. The area of the CV curves also grew as the scan rate went up, indicating high electron conduction, which also raised the height of the redox peak. The contour styles barely changed even at the highest scan rate (50 mV s^−1^), demonstrating that this material has swift charge transfer characteristics. At different scan speeds, Fig. 6b shows the specific capacitances of the working electrode and were computed using the subsequent Eq. (2) [72]:

2 $${\mathbf{C}}_{{\mathbf{s}}} = \smallint {\mathbf{Idv}}/{\mathbf{mn}}\Delta {\mathbf{V}}$$

where *n* is the scan rate, ∆V is the maximum potential, dv is the potential window, I is the current, and m is the mass of the electro-active material on the electrode. At scan speeds of 10, 20, 40, and 50 mV s^−1^, respectively, the specific capacitance values were 1044, 933, 822, and 773 Fg^−1^.

Further the cyclic voltammetry at higher potential window upto 1.2 V showed the current increasing trend with increase in sweep voltage, but does not provide a suitable and sufficient increment in the capacitance values. Therefore, the performance of the electrodes remains most stable at 0–0.45 V and after 3 h of continuous testing the stability of cyclic voltagram remains almost unchanged and provided almost 94% stability at a high scan rate of 50 mV/s.

### Galvanostatic charge–discharge measurements

**Fig. 7:**
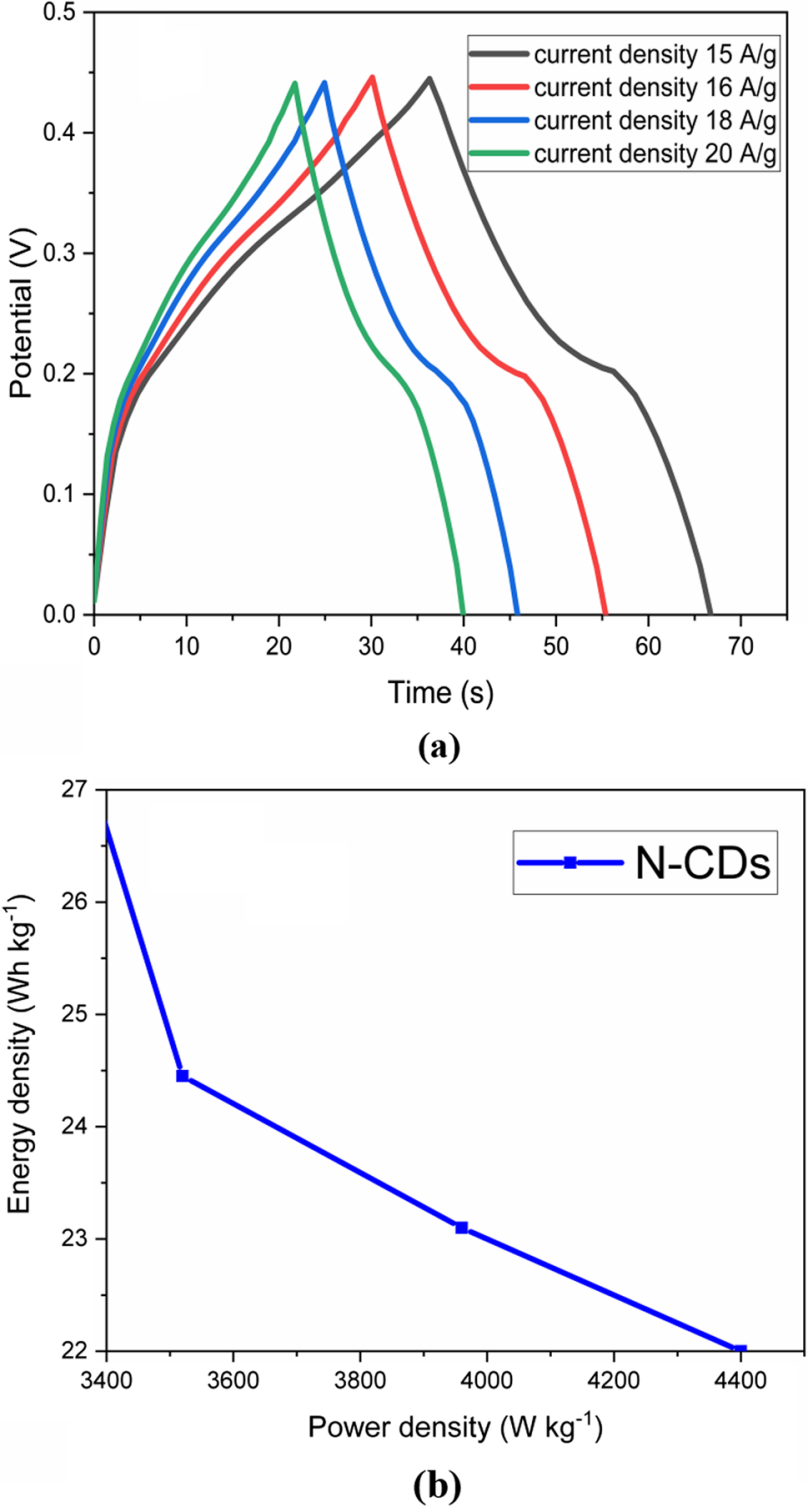
**a** galvanostatic charge discharge curves of Ni/N-CDs working electrode at different current densities. **b** Ragone plot of working electrode material

Galvanostatic charge–discharge measurements were performed in a voltage range between 0 and 0.45 V to further evaluate the electrochemical activity of the Ni/N-CDs electrode. The results are shown in Fig. 7a. The small plateaus here show battery-like characteristics. In order to compare the curves at various current densities ranging from 15 to 20 A g^−1^, the measured currents of the N-CDs electrode were adjusted in accordance with the amount of active material coated on the NF substrate. Equation (3) [72] was used to get the capacitance values:

3 $${\mathbf{C}}_{{\mathbf{s}}} = \, {\mathbf{I}} \, * \, \Delta {\mathbf{t}} \, / \, {\mathbf{m}}*\Delta {\mathbf{V}}$$

where *I* denote the discharge current, ∆t and ∆V denotes the discharge time and potential window, C_s_ denotes the specific capacity, and m denotes the mass of active material coated on the substrate.

At current densities of 15, 16, 18, and 20 A g^−1^, respectively, the Ni/N-CDs-nanocomposite's specific capacitance values have been identified to be 1011, 900, 859, and 818 F g^−1^. It appears that the Ni/N-CDs electrode has strong reversibility and rate capacity since the discharge time reduced as the current density increased and no discernible shape change was seen across the profiles at varied current densities. The Ni/N-CDs electrode's Ragone plot (power density vs. energy density) is displayed in Fig. 7b. The following Eqs. (4 and 5) [72] were used to determine the values for energy density and power density:

4 $${\mathbf{E}} = {\mathbf{C}} \, \left( {\Delta {\mathbf{V}}} \right)^{{\mathbf{2}}} /{\mathbf{2}}$$

5 $${\mathbf{P}} \, = \, {\mathbf{E}}/{\mathbf{t}}$$

where P (kW kg^−1^) is the power density, C (F g^−1^) is the total specific capacitance, E (W h kg^−1^) is the energy density of the supercapacitor electrode material, and (t) is the discharge duration (h). The energy and power density estimated between 15 and 20 Ag^−1^ for this hybrid supercapacitive electrode material were (27.81–22) Wh/kg and (3331–4400) W/kg, respectively. Therefore Ni/N-CDs hybrid is thus inexpensive and promising hybrid supercapacitive electrode material.

**Fig. 8:**
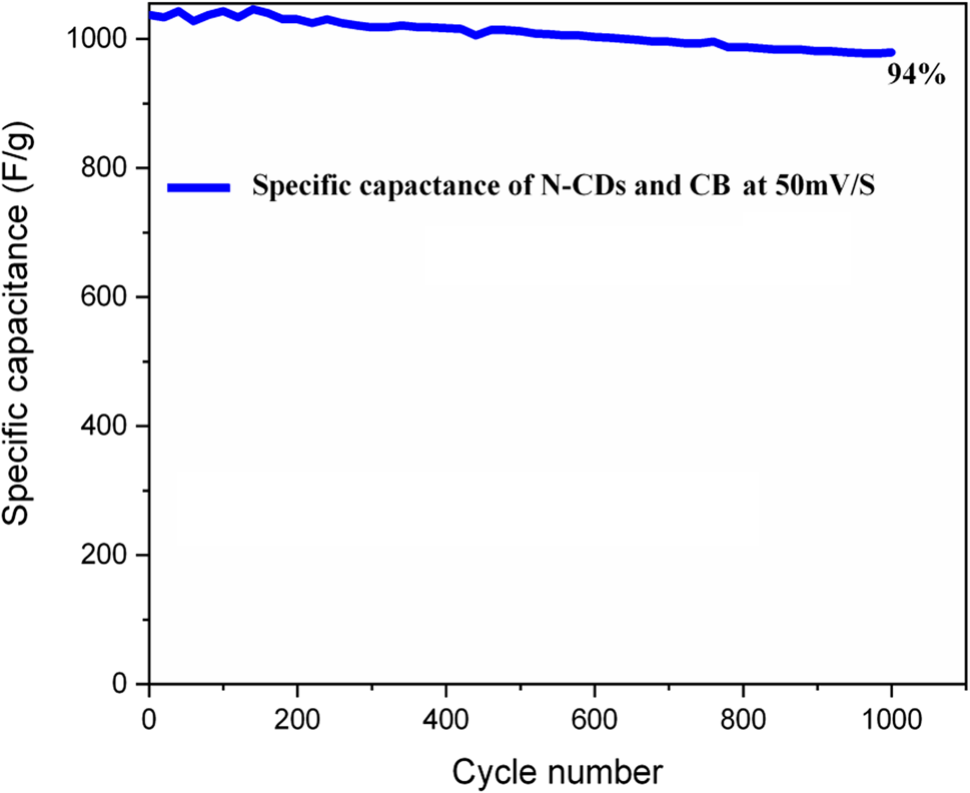
Figure demonstrating long term stability of N-CDs and CB

Furthermore, we have checked the stability of N-CDs and CB-based electrode with scan rate of 50 mV/s over 1000 cycles, within a potential window of 0–0.45 V and the overall stability remains at 94% after 1000 cycles. The capacitive values from CV against cycle numbers are shown in Fig. 8 which demonstrates the remarkable long life cyclic stability of N-CDs and CB.

### Bioimaging and antifungal activity.

**Fig. 9:**
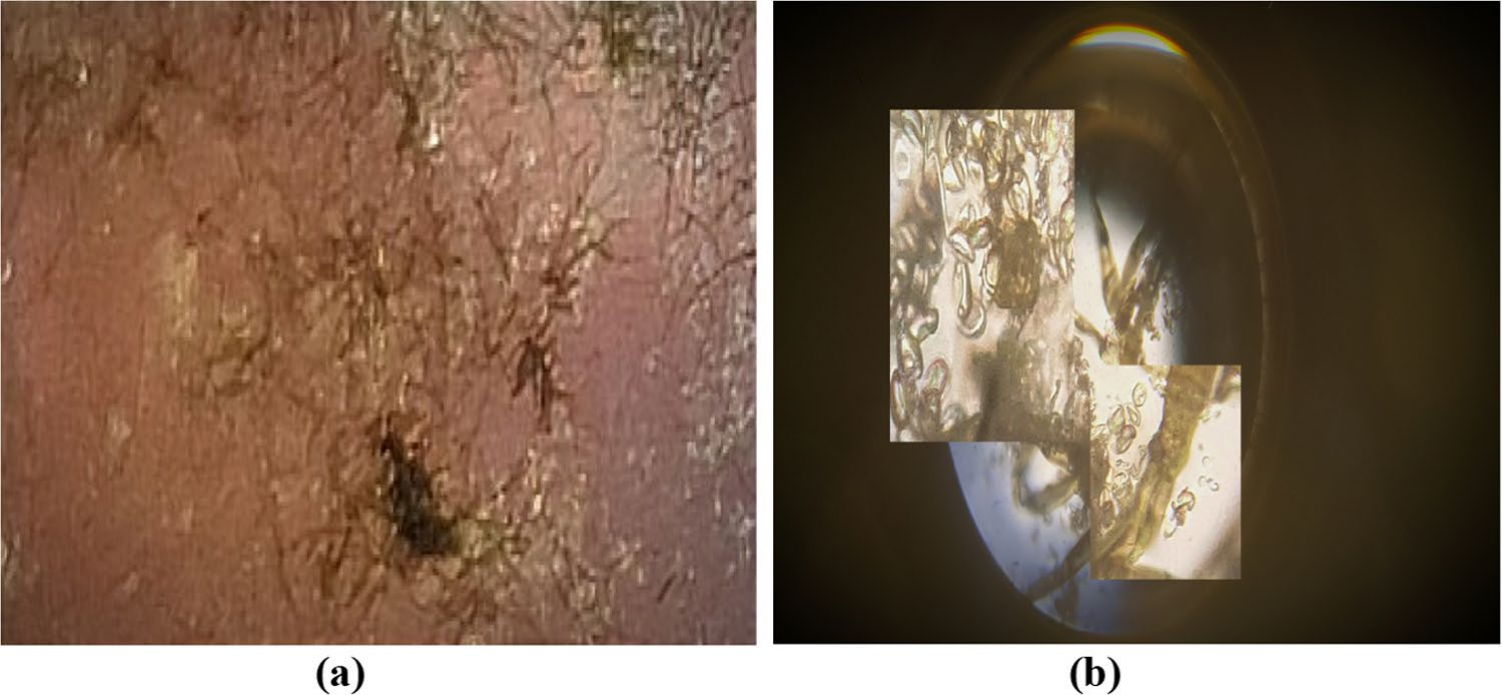
**a** culture media of Cladosporium cladosporioides mould **b** microscopic view of mould under 50 × zoom

The Cladosporium cladosporioides culture nurtured on PDA a cross-section zoomed view of that is shown in Fig. 9a which is a commonly fungus grown on agricultural products and resulting big loss to farmers and households therefore it is necessary to control such losses. Further, microscopic view of the mould without addition of heteroatom-doped N-CDs nanocomposite under white visible light at 50 × zoom is shown in Fig. 9b demonstrating fungal spores (Cladosporium-cladosporioides) as well as mycelium (hyphae), the main absorbing and transporting system of mould. The synthesised heteroatom-doped N-CDs incorporated nitrogen which existed in the bulk ratio along with small quantities of sulphur, phosphorus, potassium, magnesium, and zinc demonstrated overall P type behaviour of N-CD’s (net positive charge). These substances have moderate to strong antifungal activity, as reported by the literature against the undoped carbon dots, because undoped carbon dots possess less number of active centres and higher energy gap which makes it difficult to transfer the active species, thereby fails to bind with the charge active surface of fungal cell wall and other intercellular organelles rather than multidoped-based carbon nano-dots.

**Fig. 10:**
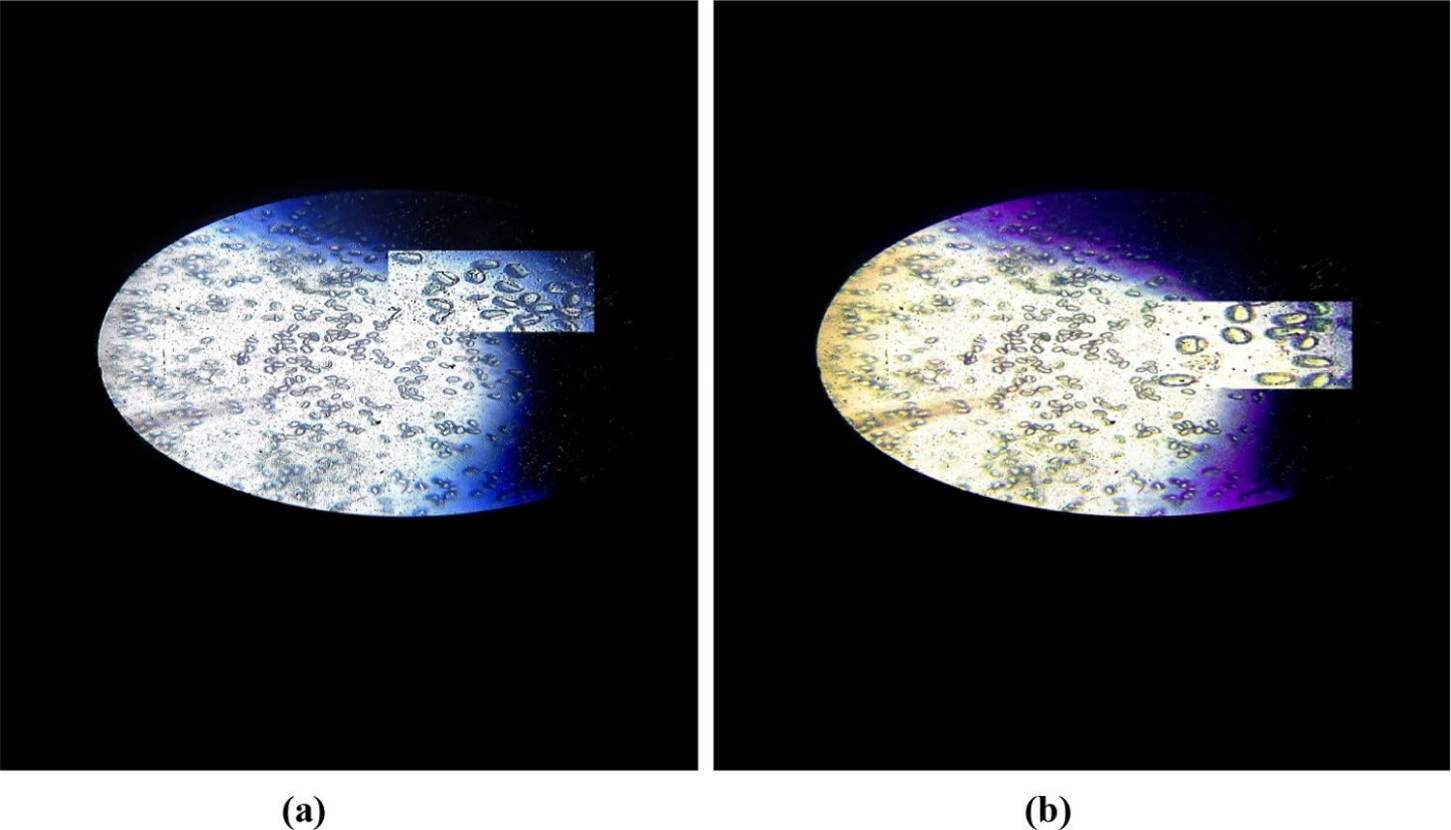
Bioimaging as well as antifungal activity of heteroatom-doped CDs nanocomposite effect on fungus after 3 h under white light (left side) and light yellow visible light (right side)

We have checked various concentrations of N-CDs but the optimised concentration of heteroatom nanocomposite, dissolved in clean water at a Weightage % of 5 g/100 ml (50 μg/ml) in a culture of the desired mould, and incubated at 25 °C for 3 h which gave the best results against imaging and antifungal process. Further the final outcome showed that the heteroatom N-CDs have a significant impact on mould culture growth due to the net P type behaviour of surface moieties (N, S, P, and Zn) of heteroatom-doped CDs nano-composites absorbed by the fungal mycelium, resulting in the formation of isocyanates that react with the thiol-groups of the cells' enzymes and metabolites to destroy the hyphae and disrupt the flow of energy to the fungal cells. Furthermore, through phagocytosis, the heteroatom-doped N-CDs reached the cytoplasm without seriously harming the fungal cell wall and accumulated rapidly in the myelin bodies and vesicles, while some of them are able to penetrate the nucleus where they form a connection with the DNA, RNA, and chromatin, degrading the nucleic material and eventually causing cellular death in the fungal strain. In addition the very famous mechanism of the P type character of N-CD’s damages the negatively charged cell wall, increases the permeability through electrostatic interactions, induces the generation of ROS and further effects the cellular mechanism, leading to cellular death and the dead cells are shown in Fig. 10a and b and without any stainer. That demonstrated the fact that the N-CDs have entered the cells and killed the cells, finally presence of fluorescent N-CDs inside and on the surface of cells and gives us the clear and sharp fluorescent imaging of these cells under visible light (white and light yellow) as illustrated in Fig. 10a and b without any stainer.

## Conclusion

In this study, we investigated a microwave irradiation-based green method for creating heteroatom-doped N-carbon dots from pumpkin seeds. Numerous characterisation techniques were utilised, including scanning electron microscopy (SEM), Fourier transform infrared spectroscopy (FT-IR), X-ray diffraction (XRD), energy dispersive X-ray spectroscopy (EDX), cyclic voltammetry (CV), UV–Vis absorption, and fluorescence spectroscopy. This one-step synthesis technique has various benefits such as quick production, efficient, affordable, environmentally friendly, simple to create, and exhibiting an outstanding fluorescence quantum yield of up to 65.50%. Herein, the Ni/N-CDs working electrode material exhibited a significant supercapacitance value of 1044 F/g^−1^ with a scan rate of 10 mV/s and a high energy density of 28.50 Wh/kg corresponding to a power density of 3350 W/kg and clarified the high density energy storage characteristics of N-CDs. Furthermore, the N-CDs showed significant antifungal and bioimaging activities against Cladosporium cladosporioides mould sample.

## Data Availability

The datasets generated during and/or analysed during the current study are available from the corresponding author on reasonable request.
